# Hikikomori in the Middle East: The role of problematic gaming, social media use, and loneliness

**DOI:** 10.1371/journal.pone.0312818

**Published:** 2024-10-29

**Authors:** Harshil Shah, Mai Helmy, Zahir Vally

**Affiliations:** 1 Department of Clinical Psychology, United Arab Emirates University, Al Ain, United Arab Emirates; 2 Department of Psychology, Sultan Qaboos University, Muscat, Sultanate of Oman; RIKEN CBS: RIKEN Noshinkei Kagaku Kenkyu Center, JAPAN

## Abstract

Hikikomori, a form of severe social withdrawal has been found to be associated with behavioral addictions such as gaming addiction and problematic social media use (PSMU). Since literature related to hikikomori is lacking in the Middle East, this study aimed to determine whether there are significant differences in hikikomori-like traits between two different types of social media users and gamers and investigate loneliness as a potential mediator between hikikomori and the two types of problematic behaviors. A cross-sectional, correlational design was employed, collecting a final sample of 220 participants residing in Middle Eastern countries using a mixture of convenience and snowball sampling. Results showed that passive social media users demonstrated significantly greater hikikomori-like traits compared to active users. Furthermore, hikikomori-like traits exhibited significant positive associations with problematic gaming, PSMU, and loneliness. Two separate mediation analyses, the first with PSMU and a second with problematic gaming as predictors, revealed that loneliness acted as a significant mediator between both these problematic behaviors and hikikomori-like traits.

## Introduction

Hikikomori, once thought to be a Japanese culture bound syndrome [1], has now been observed and studied across the world in both developed and developing countries alike. Cases have been reported in the Middle East as well, particularly in Iran [2], Turkey [3] and Oman [4, 5], as well as from other parts of the world, such as the USA [6], several countries in Europe, such as Spain [7], France [8], Poland [9], Ukraine [10], and countries in East and South Asia like India [2], Korea [11], Taiwan [2], and Hong Kong [12, 13]. However, there is a lack of research on the risk factors of hikikomori from the Middle East. This condition can result in significant economic loss due to the affected individuals being unemployed and may cause considerable psychological stress and financial burden, both on the afflicted and their immediate family. One study showed that individuals reported a significant negative impact of hikikomori on their quality of life [14]. Another study reported several negative effects on the physical health of people suffering from hikikomori such as high blood pressure, obesity, and hypertension [15]. This is to be expected as socially withdrawn individuals do not venture out of their homes and sometimes even their rooms, which combined with a lack of exercise, results in adverse outcomes for the individual in terms of their physical health and wellbeing. Additionally, it has been associated with various psychological disorders which can increase the burden on the mental health infrastructure and services of a country. Some of these disorders include depression [16], autism [17, 18], schizophrenia [19], social anxiety disorder [20], and behavioral addictions such as problematic gaming and social media use [21]. Therefore, it is imperative that researchers study the prevalence and risk factors associated with hikikomori to inform treatment and understand this syndrome from the Middle Eastern perspective. This evidence points to hikikomori being a pernicious and ubiquitous phenomenon that deserves immediate attention from researchers globally, especially in the Middle East due to the paucity of literature concerning this phenomenon.

The United Arab Emirates (UAE) has a culturally, linguistically, and ethnically diverse population of almost 9.3 million people and a Gross Domestic Product (GDP) of 507 billion USD [22, 23]. The country, due to rapid economic growth and development after the discovery of oil and gas has seen many changes in its demographics and culture, from being a country of fishing villages and oasis towns in the 1970s to becoming a prosperous and robust economy in the 21^st^ century [24]. According to the World Bank [25], the UAE is classified as a high income country with a GDP per capita of 53,708 USD. To date, there has been no study on the prevalence and risk factors of hikikomori in the country, and while there has been a study in Oman, a country similar to the UAE, the sample was obtained during the COVID-19 pandemic which may have resulted in biased results and an inflated prevalence rate of over 40% [4].

As people who exhibit hikikomori-like traits are socially withdrawn, it is worthwhile to explore avenues through which they may be able to interact with others without leaving their homes and receive social support. A study from Hong Kong reported that the quality of life of socially withdrawn youth increases with the length of their social withdrawal [12]. The same study also showed that as their social withdrawal progressed, they became more involved with online activities and support groups where they could interact with others like them over the internet. In a similar study, it was reported that socially withdrawn youth can develop relationships with others online, with friendships formed on virtual platforms having a higher degree of intimacy compared to offline friendships [13]. These studies point to the possibly healthy role social networking sites (SNS) may play in developing strong bonds and social support online for people with hikikomori. However, social media use can be addictive and could be an unhealthy coping mechanism for socially withdrawn people. In a study by Stavropoulos et al. [26], the participants who were at high risk of hikikomori were more likely to show problematic smartphone usage. While smartphone addiction and social media addiction may be different issues, they are related as studies have shown that smartphone addiction is a significant predictor of problematic social media use (PSMU) which results in several negative outcomes such as fear of missing out and poor academic performance [27]. PSMU may also expose people with hikikomori to online bullying which may further alienate them from others and aggravate their symptoms of social withdrawal [28]. Therefore, SNS and PSMU may play a role in predicting hikikomori -like traits.

Gaming could represent another way of coping for people with hikikomori. In a two-group comparison study [21], results showed that Japanese participants who used the internet primarily for gaming were more likely to show hikikomori symptoms. A study that obtained American and Australian samples also reported a positive association between hikikomori symptoms and scores on the Internet Gaming Disorder (IGD) scale, with longer time spent on gaming, strengthening the association between IGD and hikikomori [26]. Nevertheless, there is a debate as to whether the hikikomori condition results in people developing IGD or IGD being one of the causes of hikikomori [29]. There is also a lack of research differentiating between online and offline gaming types and examining whether offline gaming is related to adverse outcomes as well, even though the eleventh edition of the International Statistical Classification of Diseases and Related Health Problems (ICD-11) discriminates between the two types [30]. One study revealed that online gamers report spending more hours on gaming and having more maladaptive cognitions than offline gamers [31], however, more research is needed to ascertain whether offline gaming is indeed less harmful and unrelated to social withdrawal, particularly in the Middle Eastern context, and to what extent.

Problematic gaming has been found to cause loneliness due to the increased importance given to gaming resulting in the deterioration of relationships with others [32]. During the COVID-19 pandemic, because of the lockdowns, both non-clinical gaming and IGD were related with loneliness, as gaming was potentially used as a coping mechanism to deal with feeling alienated [33]. Loneliness has also been associated with PSMU, particularly passive use of social media. Passive use is marked by browsing or scrolling through the apps or SNS without contributing or sharing anything, whereas active use includes interacting with others and posting various forms of media online. One study using ecological momentary analysis found that passive social media use is related to depressive symptoms including loneliness, indicating that feeling lonely may cause individuals to browse SNS passively [34], with another study from Iceland showing a relation with depression and anxiety symptoms and passive use in adolescents [35]. Finally, people with hikikomori tend to report high levels of loneliness and a lack of social support in several countries around the world [36, 37]. There is however a dearth of studies investigating the role of loneliness as a mediating variable between gaming and hikikomori as well as PSMU and hikikomori.

Keeping the above literature review concerning the hikikomori phenomenon in mind, we proposed the following a priori hypotheses:

H1: Hikikomori-like traits will be significantly higher for individuals using social media passively as opposed to active users.
H2: Hikikomori-like traits will be significantly higher for online gamers as opposed to offline gamers.
H3: It is predicted that the following risk factors: disordered gaming, PMSU, and loneliness, will be significantly and positively associated with hikikomori-like traits.
H4: Loneliness will significantly mediate the relationship between hikikomori-like traits and gaming.
H5: Loneliness will significantly mediate the relationship between hikikomori-like traits and PMSU.

## Methods

### Research design

This was a cross-sectional study. The dependent variable was the hikikomori-like traits and the independent variables were demographics, problematic gaming, PSMU, and loneliness. The role of loneliness was also investigated as a mediator between problematic gaming and hikikomori traits, and PSMU and hikikomori traits.

### Data collection

Data was collected by circulating the survey in different classes in various departments including students from social sciences, engineering, natural sciences, and mathematics. Public spaces at the university such as the library, cafeteria, dorms, etc. where the researcher would introduce the study to willing participants, and they would scan a QR code allowing them to access the questionnaire. Students were also encouraged to share the survey link with their colleagues via online messaging applications thus incorporating snowball methods of data collection. Besides university grounds, the questionnaire was also disseminated on online websites, instant messaging applications, social media sites, and online groups used by students for wider reach. The survey questionnaire was entirely in English since the university of the primary investigator was an English medium institution and therefore the students were expected to be proficient in the language.

### Ethics

Recruitment took place from 19 January and concluded on 6 March 2024. Before the participant could access the questionnaire, they were informed about the objective of the study and their rights as a participant, in a consent form along with a brief description of the study aims and the researcher’s email for any questions or suggestions. No identifying information (student ID, name, etc.) was collected from the participants, ensuring anonymity. Participants provided written informed consent. Ethical approval from the university’s Social Sciences Research Ethics Committee was obtained prior to conducting the study (reference number: ERSC_2023_3932).

### Participants

The target population of this study were individuals residing in the Middle East aged 18 years and older. Demographic details such as participant’s age, gender, education level, socio-economic status and nationality were collected.

As this study investigated loneliness as a mediating variable, the number of participants was determined by Monte Carlo Power Analysis for Indirect Effects [38]. The web version of the application was used (https://schoemanna.shinyapps.io/mc_power_med/), and by setting the power to .8 and assuming medium effect sizes of .3 for the relationship between all three paths, the ideal sample size was calculated to be 112.

### Materials

#### Demographics

The questionnaire included questions for the following demographic characteristics: age, gender, educational level, country of origin, and employment status.

#### Hikikomori Questionnaire (HQ-25)

The HQ-25 was developed to measure the intensity of symptoms related to hikikomori over the preceding six months [39]. It is a self-administered questionnaire of 25 statements measuring symptoms of hikikomori over three subscales; namely, socialization, isolation, and emotional support. The respondents were asked how accurately the statements describe them, requiring them to answer by picking one of the five options on a Likert Scale (“Strongly disagree” = 0 to “Strongly agree” = 4) which was summed to give a total score. A cut-off score of 42 out of a maximum of 100 which has a diagnostic sensitivity of 94%, was used to determine whether the individual is at risk of hikikomori [39]. Six items of this questionnaire were reverse scored to provide a total scale score. Some examples of items include, “It is hard for me to join in on groups”, and “People bother me”. Teo et al. [39], reported a three-factor structure was found which accounted for 46.53% of the variance, the Cronbach’s alpha for the entire questionnaire was .96 and the HQ-25 had a high convergent validity with UCLA Loneliness Scale (*r =* .88, *p <* .0001). Al-Sibani et al. [4], performed exploratory factor analysis on the HQ-25 using an Omani sample with results similar to Teo et al., including a three-factor structure accounting for 52.87% of the variance, with acceptable results for the goodness-of-fit indices of the Confirmatory Factor Analysis (CFA), and an overall Cronbach’s alpha of .92, indicating this questionnaire’s applicability in the Middle East. For this sample, Cronbach’s alpha was .89.

#### Game Addiction Scale (GAS)

The GAS [40] is a tool for measuring gaming disorder (GD) symptoms. Based on the primary signs of addiction, the seven items encompass the majority of the general diagnostic criteria found in the fifth edition of the Diagnostic and Statistical Manual of Mental Disorders (DSM-5) [41], and the ICD-11 [30]. The scale measures seven factors of behavioral addictions proposed by [42], that is, salience, tolerance, mood modification, relapse, withdrawal, conflict, and problems. The participants were provided with a 5-point Likert scale, with 1 representing “never” and 5 representing “very often,” to indicate how frequently they had each symptom. “Did you think about playing a game all day long?” and “Have you felt bad when you were unable to play?” are some example questions from the scale. The 7-item version of the scale had a Cronbach’s alpha of .86 in the first sample and .81 in the second sample, and a good concurrent validity in both samples [40]. This scale can be used in a sample from the Middle East as evidenced by Asaad at al. [43], who reported good internal consistency and reliability with an alpha value of .81 and κ statistics ranging from .53 to 1 respectively. A Cronbach’s alpha value of .9 was obtained for this study, indicating excellent internal consistency.

#### Bergen Social Media Addiction Scale (BSMAS)

Bergen Social Media Addiction Scale (BSMAS) is an altered version of the Bergen Facebook Addiction Scale (BFAS) [44]. “Facebook” was substituted by “social media”, with social media being defined in the instructions as “Facebook, Twitter, Instagram, and the like.” As mentioned previously, this scale is also based on six basic components of addiction, namely, salience, conflict, mood modification, withdrawal, tolerance, and relapse. A 5-point Likert scale (ranging from “Very rarely” = 1 to “Very often” = 5) was used to measure symptoms of PSMU over the preceding year consisting of questions such as, “Used social media so much that it has had a negative impact on your job/studies?” and “Felt an urge to use social media more and more?”. The Cronbach’s alpha of the BFAS was .83 with a 3-week test-retest reliability coefficient of 0.82 and good convergent validity was reported as evidenced by correlations with other scales measuring activity on Facebook [44]. The BFAS was validated in a sample of Tunisian adolescents with a Cronbach’s alpha of .87 and significant correlations (*p* ≤ .001) with measures of video game addiction and depression [45], which suggests that this scale can be applied on a Middle Eastern sample. For the present study, this scale was internally consistent (α = .84).

#### UCLA Loneliness Scale

The original University of California Los Angeles (UCLA) Loneliness scale was constructed to be a short, 20 item, reliable, and valid tool to measure the degree of loneliness in individuals [46]. It was subsequently modified by Russel [47], in version 3 of the scale by adding “How do you feel” before every statement in the original questionnaire. Some sample items include, “How often do you feel that you lack companionship?” and “How often do you feel close to people?”. The participants were asked to respond on a 4-point Likert scale ranging from “Never” (scored as 1) to “Always” (scored as 4). Nine items in the scale were reverse scored and the sum total of the responses represent the degree of loneliness the respondent feels, with higher scores indicating increased levels of loneliness. Significant connections between the scale and other loneliness measures suggested that the scale has convergent validity [47]. Significant associations with measures of how adequate a person’s interpersonal relationships are, as well as correlations between loneliness and health and well-being indicators, all supported the construct validity of the scale [47]. Across four samples (students, nurses, teachers, and elderly), the alpha coefficient ranged from 0.89 to .94, and a test-retest coefficient of .71 in the elderly sample [47]. According to AlNajjar and Dodeen [48], the scale’s internal reliability was validated in a sample from the UAE, and the findings demonstrated that the scale accurately represented two primary components, which were split into favorably and negatively phrased questions. Excellent internal consistency was found for the present study (α = .9).

#### Type of social media use

Participants were asked to select their type of social media use by answering one question which will group them into either “active” or “passive” social media users. The question was as follows: “How do you spend most of your time on social media?”, and the two options to choose as the answer were: (a) “I spend most of my time on social media browsing and scrolling through various posts.” (“passive” use), or (b) “I spend most of my time on social media sharing and uploading or chatting with others.” (“active” use).

#### Type of game preference

Similar to grouping participants as “active” or “passive” social media users, respondents were grouped into “online” or “offline” gamers depending on whether they primarily play online or offline games. The following question was used, “What kind of games do you usually play (on any platform, be it console, PC or smartphone)?”, and the following two options were provided to the participants to choose from: (a) “Online games, that is, multiplayer games requiring an internet connection, such as Fortnite, Overwatch, League of Legends, etc.” or (b) “Offline games, that is, single-player games generally played without connecting to the internet, such as Undertale, The Witcher, God of War, etc.”.

#### Data analysis plan

Data analysis was conducted using SPSS Version 29. The dataset was first checked for missing data and mean imputation was used for one value to fill in the missing age of a participant as less than 20% of the data was missing. The descriptive statistics, including the mean and standard deviation of the continuous variables as well as the percentages of categorical demographic variables were obtained. Independent samples t-tests were used to determine whether there is a significant variation in the hikikomori-like traits in relation with type of social media use (active and passive use) and gaming preference (online and offline). Pearson’s correlation coefficients between the continuous variables (hikikomori-like traits, problematic gaming, social media use, and loneliness) were obtained as a correlation matrix to examine the potential relationships between them.

Process Macro version 4.2 (www.processmacro.org/index.html) was used for the mediation analyses with loneliness as a single mediator [49]. Two mediation models were computed, first, loneliness as a mediator between problematic gaming (predictor) and hikikomori-like traits (outcome), and second, the effects of the same mediator were investigated between problematic social media use (predictor) and hikikomori-like traits (outcome). A bootstrapping procedure (10,000 samples) was used for the mediation analyses that resulted in bootstrapped confidence intervals (95% CI). A *p* value of .05 was set to determine statistically significant results for all analyses.

## Results

### Sample statistics

A total of 229 responses were collected, however, 9 responses were removed for various reasons (3 respondents refused to provide consent, and therefore did not complete the survey, 1 respondent was over the age of 35 which was set as the exclusion criteria, 2 were below the age of 18, and 3 respondents did not provide accurate demographic details and were excluded from further analysis). Hence, the final sample consisted of 220 participants (*M*_age_ = 21.49 years, *SD* = 3.31) which was relatively evenly split between males (52.7%, *n* = 116) and females (46.4%, *n* = 102), with 2 participants preferring to not reveal their gender (Table 1). Moreover, Table 1 also shows all the other categorical and demographic variables collected from the participants. With regards to their country of origin, most of the participants were Emiratis (60.9%, *n* = 134) and the majority had completed an undergraduate degree (*n* = 96), about a quarter (26.4%) had graduated from high school, ~17% had completed a master’s degree, ~11% had completed some college or vocational training, and only a small minority of 1.8% had completed a doctoral or a post-graduate degree. The majority of the sample reported being full-time students (85%, *n =* 187), 10% were employed, and a small percentage of 2.7% and 2.3% were unemployed or employed part-time respectively. Table 2 provides a summary of all the continuous variables such as age, levels of hikikomori-like traits, and problematic behaviors in the participants.

**Table 1:** Participant characteristics.

	Variables	n (%)
**Gender**	Male	116 (52.7%)
	Female	102 (46.4%)
	Prefer not to say	2 (0.9%)
**Education**	High school graduate	58 (26.4%)
	Some college or vocational training	24 (10.9%)
	Bachelor’s degree	96 (43.6%)
	Master’s degree	38 (17.3%)
	Doctorate or professional degree	4 (1.8%)
**Employment Status**	Employed	22 (10%)
	Unemployed	6 (2.7%)
	Student	187 (85%)
	Part-time employment	5 (2.3%)
**Country of Origin**	UAE	134 (60.9%)
	Oman	31 (14.1%)
	India	14 (6.4%)
	Palestine	9 (4.1%)
	Jordan	5 (2.3%)
	Pakistan	4 (1.8%)
	Saudi Arabia	3 (1.4%)
	Sudan	3 (1.4%)
	Syria	2 (0.9%)
	Bahrain	2 (0.9%)
	Yemen	1 (0.45%)
	Libya	1 (0.45%)
	Uruguay	1 (0.45%)
	Lebanon	1 (0.45%)
	Colombia	1 (0.45%)
	Algeria	1 (0.45%)
	Ethiopia	1 (0.45%)
	Bangladesh	1 (0.45%)
	Sri Lanka	1 (0.45%)
	Kazakhstan	1 (0.45%)
	Egypt	1 (0.45%)
	Romania	1 (0.45%)
	Iraq	1 (0.45%)
**Gaming Preference**	Online	109 (49.5%)
	Offline	111 (50.5%)
**Social Media Use**	Passive	173 (78.6%)
	Active	47 (21.4%)

Data are the categorical and demographic variables with the number of participants in each category and their percentages.

**Table 2:** Mean and standard deviation of continuous variables.

Variable	*M*	*SD*
Age	21.4932	3.31369
Socialization	20.06	9.696
Isolation	14.65	5.57
Emotional Support	9.57	4.224
HQ-25	44.28	16.835
GAS	16.32	7.142
BSMAS	16.34	5.517
UCLA Loneliness Scale	50.15	11.128

Data are the mean and standard deviation of all the continuous variables. HQ25 = Hikikomori-25 Scale, GAS = Game Addiction Scale, BSMAS = Bergen Social Media Addiction Scale

### T-tests results

The sample was divided into groups based on the participant’s gaming preference and type of preferred social media use. Offline gamers were in the majority (50.5%, *n* = 111) while the rest preferred online gaming. Most of the participants reported using social media passively (78.6%, *n =* 173) and the remaining were active users who preferred to chat, share, and upload content online. There was no significant difference between hikikomori-like traits between the two types of gamers which was predicted in H2, however, in support of H1, passive users of social media (*M* = 45.5, *SD* = 16.52) reported higher hikikomori-like traits as compared to active users (*M* = 39.81, *SD* = 17.4), *t*(219) = 2.07, *p* = .04, *d* = .34, indicating a small-to-medium size effect.

### Correlation analysis

Pearson’s correlation coefficients of the scores on four scales (HQ-25, GAS, BSMAS, UCLA Loneliness Scale) and three subscales of the HQ-25 (socialization, isolation, and emotional support) were obtained (Table 3). The three subscales of the HQ-25 were significantly and positively correlated with each other and with the total HQ-25 score. HQ-25 scores were significantly and positively correlated with all the three scales to screen for addictive behaviors and loneliness with *r* values ranging from .71 to .22 as predicted by H2. There was a significant relationship between hikikomori-like traits and scores on the GAS, *r* = .38, *p* < .001. Scores on the BSMAS were correlated with HQ-25 scores, *r* = .22, *p* < .001. Finally, HQ-25 scores were correlated with subjective loneliness reported by the participants, *r* = .71, *p* < .001.

**Table 3:** Correlation analysis between all study variables.

Variables	1	2	3	4	5	6	7	8	Mean
1. Age	-								21.49
2. Socialization	-.083	-							20.06
3. Isolation	-.142*	.699**	-						14.65
4. Emotional Support	-.062	.496**	.518**	-					9.57
5. HQ-25	-.11	.932**	.863**	.708**	-				44.28
6. GAS	-.182**	.297**	.459**	.257**	.388**	-			16.32
7. BSMAS	.035	.210**	.275**	0.059	.227**	.319**	-		16.34
8. UCLA Loneliness Scale	-.047	.621**	.585**	.627**	.709**	.235**	.171*	-	50.15

Data are Pearson’s correlation coefficients for the continuous variables

*p < .01

**p < .001. HQ-25 = Hikikomori-25 Scale, GAS = Game Addiction Scale, BSMAS = Bergen Social Media Addiction Scale

### Mediation analysis

**Fig 1:**
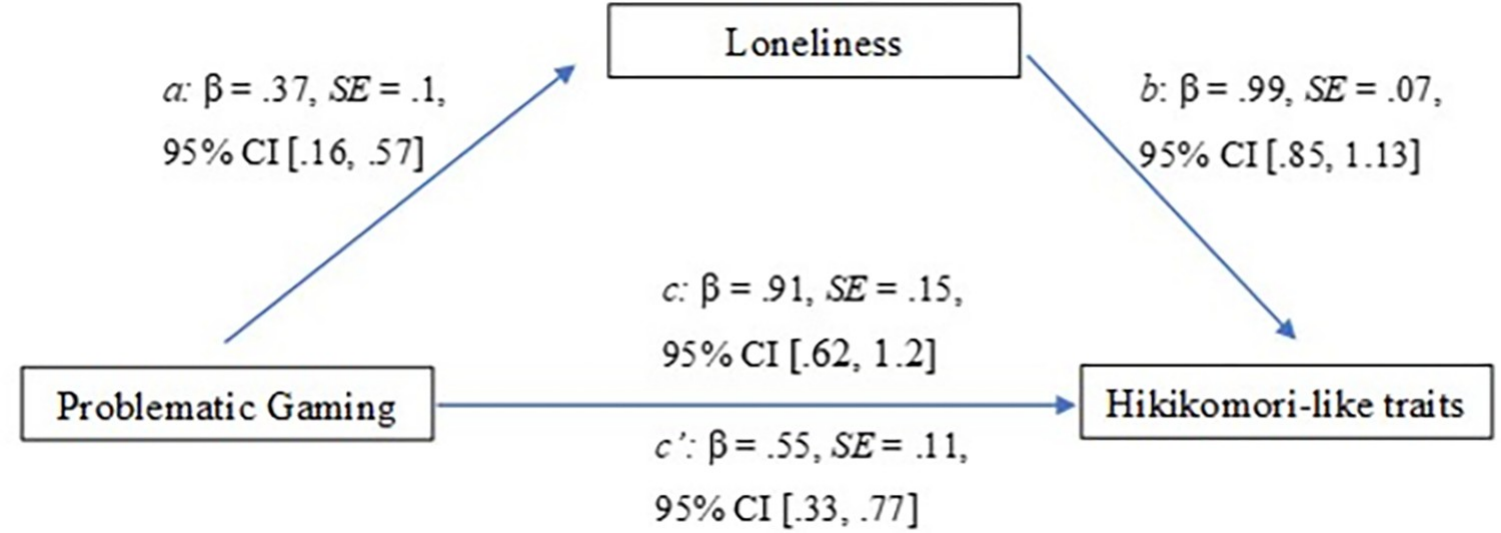
Results of the mediation analysis (problematic gaming as the predictor variable).

The following results were obtained by computing a mediation analysis of the first model (Fig 1). The overall model was significant (*F*(1, 218) = 38.47, *R*^*2*^ = .15, *p* < .001). Both paths were significant, with path *a* showing that problematic gaming was significantly related with loneliness (β = .37, *SE* = .1, 95%CI .16, .57), and path *b* showing that loneliness was significantly associated with hikikomori-like traits (β = .99, *SE* = .07, 95%CI .85, 1.13). Additionally, the total effect of problematic gaming on hikikomori-like traits was also significant (β = .91, *SE* = .14, 95%CI .62, 1.2) and this association remained as such when the indirect effects of the mediator was examined. Loneliness was determined to be a significant mediator of the relationship between problematic gaming and hikikomori-like traits (path *ab*) (β = .36, *SE* = .1, 95%CI .16, .57).

**Fig 2:**
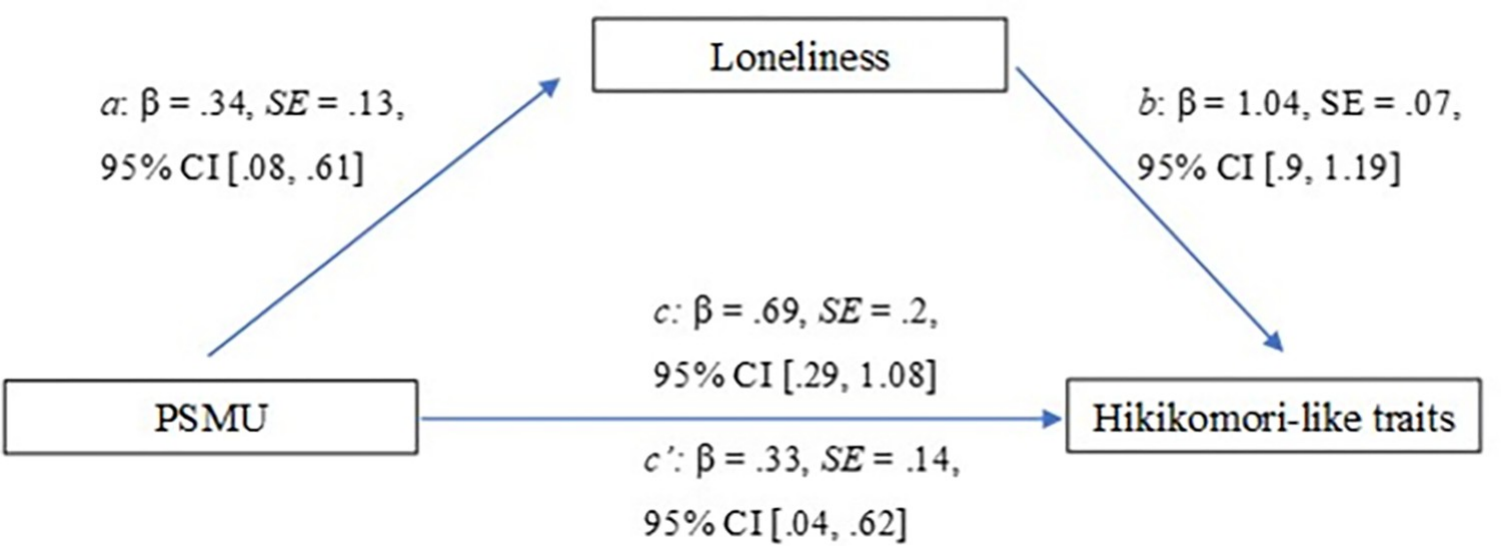
Results of the mediation analysis (problematic social media use as the predictor variable).

Similar results were obtained for the second mediation model (Fig 2), investigating the role of loneliness as a mediator between PSMU and hikikomori-like traits, with the overall model being significant (*F*(1, 218) = 11.79, *R*^*2*^ = .05, *p* < .001) and both path *a* and path *b* being significant. PSMU was significantly associated with loneliness (path *a*) (β = .34, *SE* = .13, 95%CI .08, .61) and loneliness was significantly associated with hikikomori-like traits (path *b*) (β = 1.04, *SE* = .07, 95%CI .9, 1.19). Also, total effect was found to be significant (β = .69, *SE* = .2, 95%CI .29, 1.09), remaining significant after the indirect effect of the mediator was examined. Moreover, path *ab* which represented the indirect effect in the model, showed that loneliness significantly mediated the relationship between PSMU and hikikomori-like traits (β = .35, *SE* = .14, 95%CI .07, .65).

## Discussion

Hikikomori was once thought to be a phenomenon unique to Japan but has now been found around the world. The aim of this study was twofold: one, to examine whether there are significant differences in hikikomori-like traits seen in types of gamers and social media users; and two, to examine the various risk factors, specifically, problematic gaming, PSMU, and loneliness, and their association with hikikomori-like traits, with loneliness as a potential mediator between the two behavioral addictions and hikikomori.

This study investigated the relationship between participants’ engagement with different types of games and social media and their scores on the HQ-25 scale, which measures hikikomori-like traits. Hypothesis 1 posited that passive social media users would exhibit higher levels of hikikomori-like traits compared to active users, and this was supported by the findings. Passive users scored significantly higher on the HQ-25, indicating a small-to-medium effect size. This aligns with previous research from other parts of the world suggesting a link between loneliness and passive social media use, with potential causal implications [34]. Given the association between loneliness and hikikomori [36], these results contribute to our understanding of the phenomenon by highlighting the distinct traits observed in passive versus active social media users. Passive usage, characterized by minimal interaction, may exacerbate social withdrawal symptoms, consistent with findings linking problematic media use in American university students, including social networking, to social withdrawal [50]. While online interactions can potentially benefit socially withdrawn individuals, passive browsing without active engagement may hinder such opportunities for connection and support [12, 13].

Hypothesis 2 was unsupported, as no significant difference in hikikomori-like traits was found between online and offline gamers. While the link between gaming and hikikomori has been explored in samples from Japan, Australia, and the US [21, 26, 29], prior studies focused solely on internet-based gaming without distinguishing game types. Though not hikikomori-specific, Bodi et al. [31], found online gamers reported more maladaptive cognitions and longer playtimes, which is associated with social withdrawal [26]. It was thus hypothesized that online gamers would exhibit higher hikikomori-like traits, yet this was not observed, challenging the notion that gaming type matters, at least in a Middle Eastern sample. This may also suggest that there is a need to explore gaming genres further, as specific sub-genres may be more relevant to hikikomori-like traits.

The third hypothesis predicted that disordered gaming, PSMU, and loneliness would be significantly and positively related with hikikomori-like traits. Behavioral addictions, particularly those related with technology and the internet, such as problematic gaming and PSMU, were found to be related with the hikikomori syndrome in East Asian and American samples [21, 26]. Loneliness, which is another characteristic commonly found among socially withdrawn individuals [37], was also found to be associated with hikikomori-like traits in this study. Previous research from Taiwan also indicates that individuals who are socially withdrawn report higher perceived disconnectedness with their peers, feel more lonely, and have a higher likelihood of being diagnosed with psychiatric disorders [51]. Therefore, the findings of this study support the existing view of hikikomori-like traits being significantly and positively related to problematic gaming, PSMU, and loneliness with *r* values obtained in the present research ranging from .22 to .71, thereby supporting hypothesis 3.

Another aim of this study was to explore loneliness as a mediator between addictive behaviors and hikikomori-like traits. The initial model revealed that loneliness significantly mediated the link between problematic gaming and hikikomori-like traits, supporting hypothesis 4. This finding suggests that individuals with high levels of loneliness, particularly in the context of problematic gaming, exhibit elevated hikikomori-like traits. This aligns with existing research indicating loneliness mediates the relationship between video game consumption and various negative outcomes, including sleep disturbances and relational difficulties [52]. Moreover, loneliness was predictive of problematic gaming behavior in a study from Italy [53], and was associated with both engaged and addictive gaming in a Swedish sample [54]. This supports the compensatory media usage model proposed by Kardefelt-Winther [55], which suggests that media consumption can serve as a coping mechanism, albeit less effective for psychosocially vulnerable individuals. Thus, gaming may function as a coping strategy for lonely individuals, potentially leading to the detrimental outcome of social withdrawal.

The second mediation model between PSMU and hikikomori-like traits with loneliness as a single mediator was also found to be significant, thereby supporting hypothesis 5. Preceding literature from the US supports this result as PSMU reduces the amount of social support received in real-life which is associated with depression anxiety and social isolation [56]. Moreover, in a study of Finnish social media users, high levels of PSMU predicted elevated levels of loneliness, which in turn led to negative outcomes such as decreased life satisfaction [57]. This result is also in agreement with the compensatory media use model [55], showing that social media can be used as a tool to cope with stressful conditions such as loneliness and that individuals experiencing loneliness within the context of PSMU are more likely to exhibit hikikomori-like traits.

### Clinical implications

The results obtained underscored the role of social media, particularly the negative impact of its passive use. Individuals with higher levels of hikikomori-like traits due to their social isolation may interact less with other individuals online on social media instead of using it to increase social support and develop new relationships. According to the rich-get-richer hypothesis of social media, individuals with the highest levels of current social support, a preference for social connections, no issues in social relationships, and extraverted traits would gain the most by using social media in the form of increased social capital, while individuals with introverted traits struggle to do the same [58, 59]. However, social media can have advantages, notably, it serves as a facilitator of social interactions, particularly benefiting young adults grappling with mental health challenges by providing users access to peer support [60]. This should motivate researchers to devise online interventions tailored to address the various facets of hikikomori. Interventions focusing on mitigating loneliness and social anxiety have already been formulated and have exhibited efficacy in ameliorating these adverse symptoms and enhancing overall well-being [61, 62]. Consequently, individuals exhibiting traits reminiscent of hikikomori stand to benefit significantly from social media engagement that is active in nature.

PSMU and problematic gaming showed significant positive associations with hikikomori, suggesting the potential value of interventions targeting these behavioral addictions in hikikomori individuals. Given the significant links between hikikomori-like traits and problematic SNS and video game use, interventions could utilize these platforms to connect with affected individuals and offer psychological support. Tateno et al. [63], suggested that games like Pokémon Go, which encourage outdoor activity, might benefit hikikomoris unable to leave their rooms, with another study reporting a hikikomori case that showed improvement in venturing out to collect Pokémon [64]. Such games could complement other interventions like Animal Assisted Therapy [65], psychoeducation for caretakers [66], and family training [67]. Additionally, interventions targeting problematic gaming and PSMU could be adapted for socially withdrawn youth. Cognitive Behavioral Therapy showed promise in addressing internet and computer game addiction [68], while multi-family group therapy was effective in reducing internet addiction among adolescents [69]. Online self-help interventions for social media addiction, designed for university students, could also be applicable [70, 71]. Furthermore, novel interventions tailored for socially withdrawn individuals with behavioral addictions warrant further exploration and development.

Mediation analyses revealed loneliness as a significant mediator between both addictive behaviors and hikikomori, suggesting interventions to alleviate loneliness could help prevent individuals with PSMU and disordered gaming from becoming socially withdrawn. Stice et al. [72] found that a cognitive-behavioral intervention effectively reduced depressive symptoms, including loneliness. In a sample of socially isolated adults with psychological disorders, Groups 4 Health, a newly developed loneliness intervention, significantly reduced loneliness, social anxiety, and fostered a greater sense of belonging compared to treatment as usual [73]. Additionally, a social skills intervention for young adults with autism spectrum disorder, often comorbid with hikikomori [17, 18], successfully reduced loneliness and enhanced social skills knowledge, with caregivers noting improved social responsiveness and empathy [74]. Therefore, psychological interventions targeting loneliness may be utilized for socially withdrawn young adults and future researchers should develop interventions that target not only one, but a confluence of risk factors of hikikomori.

## Conclusions

This study provides insight into the hikikomori phenomenon within the Middle Eastern context, revealing correlations between hikikomori-like traits and problematic gaming, as well as problematic social media use, particularly passive engagement, and loneliness. These results were similar to those found in populations in the West and East Asia. This suggests that interventions targeting social media usage or gaming could mitigate social withdrawal tendencies. Active engagement with social media platforms may foster positive outcomes for individuals with hikikomori, potentially enhancing their social support networks, while passive usage should be discouraged. Moreover, these findings suggest that SNS or online gaming platforms not only serve as recruitment avenues for hikikomori research but could also be adapted to facilitate the integration of socially withdrawn youth into society and potentially alleviate their isolation. Given the significant role of loneliness as a mediator between behavioral addictions and hikikomori-like traits, interventions addressing loneliness may effectively alleviate hikikomori symptoms. Further exploration in this field is imperative, with a focus on developing treatments that target not only social withdrawal but also the interplay between behavioral addictions, loneliness, and hikikomori.

### Limitations and future directions

This was a cross-sectional study, and therefore, the limitation of drawing conclusions related to cause and effect applies. Future researchers should focus on longitudinal studies since researchers have yet to ascertain the directionality of addictive behaviors related to internet use and their relationship with hikikomori [29]. The sampling methods used limit the study findings since convenience and snowball sampling were employed, obtaining a sample primarily from full-time university students. Since hikikomori involves a prolonged period of social withdrawal, the participants who were full-time students attending university may not be classified as hikikomori and may be less likely to exhibit hikikomori-like traits. Hence, a more representative sample should be obtained that accurately reflects the Middle Eastern population.

Factors other than behavioral addiction and loneliness should also be investigated, such as those hypothesized in the model proposed by Kato et al. [37], including the role of parental figures, *amae*, bullying, and socioeconomic factors [75], which may contribute to the development of hikikomori in adolescents and adults alike. This will contribute to formulating a model of hikikomori that is contextual to the Middle East and the Arab society. Moreover, Emirati families are typically patriarchal in nature with males having more freedom as compared to their female relatives, who spend a majority of their time within the confines of their homes and require permission from their male relatives should they wish to travel or work outside their homes [76]. As one of the primary features of hikikomori is physical isolation in one’s home [77], this may place Emirati females at a higher risk of becoming socially withdrawn. Future research should investigate this condition and whether there is a connection between gender and hikikomori in the Middle East.

In both the mediation models, loneliness was the singular mediator, however, the hikikomori construct is highly complex with a constellation of factors and characteristics such as time spent on SNS and gaming that could mediate the association between social withdrawal and several other problematic outcomes [26], which was another major limitation of this study. Other potential mediators that could help explain the relationship between addictive behaviors and hikikomori-like traits are attachment [78], co-morbid psychological disorders [7, 36], and interpersonal difficulties [79]. The two problematic behaviors (PSMU, and problematic gaming) may also serve as mediators since, as mentioned previously, many of the causal relationships between the variables remain unclear. For example, PSMU may mediate the association between problematic gaming and hikikomori-like traits. So, there is a possibility that the position of the variables in the models used in this study may be switched and may still produce a significant effect. For instance, hikikomori-like traits may have a causal relationship with problematic gaming and PSMU.

The study used an English questionnaire which may have affected the validity of the results. For example, while the BSMAS has now been applied in Arabic-speaking samples [80], the scale was originally designed to gauge Facebook usage and the reliability and validity of its use in this population continues to be unclear. Ideally, questionnaires and scales validated in the UAE should be used, and more scales should be translated and validated for the Emirati population. Additionally, the HQ-25 scale was designed to be a screening tool to determine whether an individual is at high risk of hikikomori and should not be used in isolation to ascertain the presence of social withdrawal [39]. Therefore, other measures such as the duration of social isolation at home, the NEET-Hikikomori Risk Factors [81], or the Hikikomori Diagnostic Evaluation [82], should be used to measure this construct, its prevalence in a population, and its risk factors.

## Supporting information

S1 File Complete dataset for the study (SPSS datafile). Dataset on which the study’s analyses are based.
(XLSX)
